# Immunoglobulin Y Specific for SARS-CoV-2 Spike Protein Subunits Effectively Neutralizes SARS-CoV-2 Infectivity and Ameliorates Disease Manifestations In Vivo

**DOI:** 10.3390/biomedicines10112774

**Published:** 2022-11-01

**Authors:** Chia-Tsui Yeh, Chia-Ying Lee, Yi-Jung Ho, Sin-An Chen, Liang-Yu Chen, Ping-Cheng Liu, Yuan-Fan Chin, An-Yu Chen, Po-Shiuan Hsieh, Yi-Jen Hung, Cheng-Cheung Chen, Yu-Chie Wang, Guan-Chiun Lee

**Affiliations:** 1 School of Life Science, National Taiwan Normal University, Taipei 116, Taiwan; 2 Institute of Preventive Medicine, National Defense Medical Center, New Taipei 237, Taiwan; 3 School of Pharmacy and Graduate Institute of Pharmacy, National Defense Medical Center, Taipei 114, Taiwan; 4 Graduate Institute of Medical Sciences, National Defense Medical Center, Taipei 114, Taiwan

**Keywords:** immunoglobulin y, yolk, severe acute respiratory syndrome coronavirus 2, spike protein, receptor-binding domain

## Abstract

(Background) The coronavirus disease 2019 (COVID-19) that is caused by severe acute respiratory syndrome coronavirus 2 (SARS-CoV-2) carries high infectivity and mortality. Efficient intervention strategies are urgently needed. Avian immunoglobulin Y (IgY) showed efficacy against viral infection whereas the in vivo efficacy remains unclear. (Methods) We immunized laying hens with S1, S1 receptor-binding domain (S1-RBD), or S2 subunits of the SARS-CoV-2 spike (S) protein. After immunization, IgYs were collected and extracted from the egg yolks. The neutralization potential of IgYs was examined by the plaque reduction neutralization test (PRNT). The bioutility of IgYs was examined in Syrian hamsters in vivo. (Results) IgYs exhibited typical banding patterns in SDS-PAGE and Western blot and were immunoreactive against S1, S1-RBD, and S2 subunits. The plaque reduction neutralization test (PRNT) showed that all purified IgYs potently neutralized different SARS-CoV-2 strains in vitro. In Syrian hamsters, the combination of IgYs for S1-RBD and S2 subunits administered before or after SARS-CoV-2 infection effectively restored body weight loss and reduced intrapulmonary lesions and the amount of immunoreactive N protein-positive cells, which were caused by SARS-CoV-2 infection. (Conclusions) Collectively, IgYs specific for S protein subunits effectively neutralized SARS-CoV-2 in vitro and in vivo and may serve as prophylactic or therapeutic antibodies in the prevention or treatment of COVID-19.

## 1. Introduction

More than two years after the outbreak of the coronavirus disease 2019 (COVID-19), the World Health Organization (WHO) has documented approximately 500 million confirmed cases and more than 6 million deaths. This pandemic is characterized by the high infectivity of the virus and associated mortality and has greatly impacted the global economy and society [1]. Severe acute respiratory syndrome coronavirus 2 (SARS-CoV-2) is a highly transmissible and pathogenic virus of the coronavirus family that causes COVID-19. This pathogen is widely accepted to predominantly infect the respiratory system, resulting in symptoms ranging from respiratory tract infection to severe pneumonia and acute respiratory distress syndrome [2]. However, several reports revealed that SARS-CoV-2 also infects extrapulmonary organs [3,4], including the heart [5], brain [6], and liver [7]. In line with these observations, the primary SARS-CoV-2 receptor, angiotensin-converting enzyme-II (ACE2), and the transmembrane protease serine type 2 (TMPRSS2) receptor have been detected in various extrapulmonary organs [3,4,5,6,7]. The entry of SARS-CoV-2 into the host cells via the cell membrane is mediated through the binding of the viral homotrimeric spike (S) protein to ACE2 and subsequent priming by TMPRSS2 [8].

Scientists have employed different innovative approaches and technologies to develop novel therapeutics against SARS-CoV-2 infection. Nevertheless, despite the immense global effort to combat SARS-CoV-2 infection, the pandemic remains poorly controlled and several SARS-CoV-2 variants have been consecutively verified. These variants include the Wuhan variant, Alpha (α) variant, Delta (δ) variant, and Omicron (ο) variant [9]. These strains have replaced the Wuhan strain, which was the first to be identified but has gradually disappeared. Among these variants, the Omicron (ο) variant is the most recently identified SARS-CoV-2 variant and has become the dominant strain globally [10]. Unexpectedly, the development of vaccines and therapeutics remains challenging and could not completely prevent SARS-CoV-2 from spreading.

To respond to the current challenge of effectively controlling and managing the pandemic, several innovative approaches have been designed and tested. Passive immunization [11] utilizes antibodies from the serum of immunized animals, immunized humans [12], and convalescent patients [13]. The generation of monoclonal antibodies was considered to be the best choice due to their high specificity. However, their high production costs may limit their development and availability [14], and this production cost may be even higher for respiratory infections due to the demand for additional antibodies [15]. The use of polyclonal antibodies has multiple hurdles including the safety of patients and standardization. In recent years, immunoglobulin Y (IgY) has drawn remarkable attention from the field and has become an interesting approach to produce inexpensive and bioavailable antibodies in large quantities [16]. IgY has similar functions to mammalian IgGs and serves as the primary circulating antibody isotype in birds, reptiles, and amphibians [17]. In addition, IgYs are transferred to egg yolks in high concentrations and are eventually passed to developing embryos to provide passive immunization [18,19]. High-yield production of IgY has shown promising potential for both prophylaxis and the treatment of bacterial and viral infections [20,21,22,23,24]. Collectively, these findings demonstrate that IgY-based technologies hold great promise in biomedical research, diagnostics, and therapeutics. Since IgYs can be obtained easily and reliably in large amounts from the egg yolks of immunized chickens in a non-invasive and cost-effective way, it remains an interesting question whether IgY may represent an alternative and optional antibody with efficacy equivalent to IgGs in clinical uses.

Considering the advantages of IgYs in biomedical applications, scientists have proposed a new hypothesis that passive immunization with IgYs may represent a potential therapeutic approach for treating viral respiratory infections [25]. Recently, Artman et al. produced IgYs targeting the SARS-CoV-2 S protein and demonstrated the ability of IgYs to lower the binding affinity of SARS-CoV-2 S proteins for ACE2 and subsequently reduce viral replication in host cells [26]. In the present study, we immunized laying hens with antigens for various subunits of the SARS-CoV-2 S protein and then extracted and purified IgYs specific for the corresponding antigens from egg yolks. We evaluated whether these IgYs were able to neutralize SARS-CoV-2 infectivity in Vero E6 cells in vitro. In addition, we infected Syrian hamsters with SARS-CoV-2 and subsequently examined whether IgY administration showed efficacy in prophylaxis and treatment against SARS-CoV-2 infection in vivo. In the present study, our findings have provided evidence demonstrating the prophylactic and therapeutic utilities of IgY antibodies against SARS-CoV-2 infection in vivo.

## 2. Materials and Methods

### 2.1. Immunization of Laying Hens

Eighty micrograms of each purified SARS-CoV-2 spike protein subunit (Spike S1-His Recombinant Protein, Cat: 40591-V08B1, Spike RBD-His Recombinant Protein, Cat: 40592-V08B, or Spike S2 ECD-His Recombinant Protein, Cat: 40590-V08B (Sino Biological Inc., Beijing, China) was dissolved in 0.5 mL of normal saline solution then emulsified with 0.5 mL of Freund’s complete adjuvant (Sigma-Aldrich, St. Louis, MO, USA). The resultant emulsified product was then used for immunizing the laying hens via intramuscular injection into 5 different sites on the muscles of the thorax. Blood samples and the laying eggs were collected once per two weeks for the purification of IgY antibodies which were used for the detection of the plateau of passive immunization. Four weeks after the immunization of laying hens by the 1st injection, the antigen proteins and incomplete Freund’s adjuvant were mixed and emulsified, followed by the 2nd immunization of these hens using the same procedures. Two weeks after the final injection, eggs were harvested and stored at 4 °C. Mock IgY was produced using the same adjuvant without antigens.

### 2.2. IgY Extraction

The extraction of IgYs was conducted using our patented method for extracting IgY from egg yolks (Wang et al., United States Patent; Patent No.: US0096.05052B2 Date of Patent: 28 March 2017). The method for extracting IgY from egg yolks is conducted as follows. Overall, a buffer solution, a yolk sample, and an inorganic salt solution were used for the extraction. Briefly, the yolk sample with the buffer solution was diluted with the acetate-based buffer solution with a pH ranging from 4.6 to 5.4. The mixture was then stirred for at least 30 min at 4 °C, followed by centrifugation. After centrifugation of the mixture at 14,000× *g* for 30 min, the supernatant was collected. Thereafter, the inorganic ammonium sulfate salt solution with a concentration ranging from 0.05 M to 0.15 M and a saturation degree from 30% to 60% was added into the supernatant to salt out the IgYs. After another stirring for 30 min, the mixture was centrifuged again at 14,000× *g* for 30 min to precipitate the IgYs. The whole extraction process requires approximately 2 h.

### 2.3. Specific IgY Enzyme-Linked Immunosorbent Assay (ELISA)

To quantitatively detect IgY antibodies against the SARS-CoV-2 spike protein subunits, we used an indirect ELISA. Ninety-six-well ELISA plates (Thermo Fisher Scientific, Waltham, MA, USA) were coated for 16 h at 4 °C with purified SARS-CoV-2 spike protein subunit (Spike S1-His Recombinant Protein, Cat: 40591-V08B1, Spike RBD-His Recombinant Protein, Cat: 40592-V08B, or Spike S2 ECD-His Recombinant Protein, Cat: 40590-V08B (Sino Biological Inc., Beijing, China), diluted in carbonatebicarbonate buffer to a concentration of 1 μg/mL. The plates were washed with 0.05% PBST, incubated with a blocking buffer consisting of 3% non-fat dried milk in PBS for 1 h at room temperature, and then washed. Chicken anti-sera and purified IgY of egg yolk (10  µg/mL) after immunization of laying hens were prepared by diluting in blocking buffer, followed by 10-fold serial dilutions. These were added in triplicate to the wells and incubated for 1.5 h at 37 °C. Samples were added to corresponding wells and incubated for 1 h at room temperature. The plates were washed and incubated with 100 μL per well of HRP-conjugated goat anti-chicken IgY (1:3000, Rabbit anti-Chicken IgY, Sigma-Aldrich, St. Louis, MO, USA) for 1 h at 37 °C. After washing, 100 μL of SureBlue™ TMB 1-Component Microwell Peroxidase Substrate (SeraCare 5120-0076, LGC Clinical Diagnostics, Milford, MA, USA) was added to the wells and incubated for 5 min at room temperature. The reaction was terminated with 100 μL of TMB BlueSTOP™ Solution (SeraCare 5150-0024, LGC Clinical Diagnostics, Milford, MA, USA). Optical density (O.D.) was measured at 450 nm with subtraction of the O.D. on an ELISA reader (Molecular Devices). The endpoint titer was defined as the highest IgY dilution at which the OD450 value was ≥0.2. All experiments were performed in triplicates.

### 2.4. Cell Lines and Viruses

All the SARS-CoV-2 strains used in this study, including the SARS-CoV-2 WA strain (hCoV-19/Taiwan/4/2020), and the three variants of concern (VOCs), i.e., B.1.1.7 (hCoV-19/Taiwan/792/2020, α variant), B.1.617.2 (hCoV-19/Taiwan/1144/2021, δ variant), and Omicron BA.1 (hCoV-19/Taiwan/IPM-63/2022, ο variant) were provided by the Taiwan Centers for Disease of the Ministry of Health and Welfare. The Vero E6 cells (ATCC CRL-1586) were generally maintained in high-glucose DMEM (HyClone) which was supplemented with an antibiotic–antimycotic (Gibco) and 10% fetal bovine serum (Hyclone). For virus propagation, SARS-CoV-2 strains were propagated in Vero E6 cells cultured in high-glucose DMEM containing 2% FBS. The 2nd passage of viruses was subjected to all the experiments in this study. Viral titers were examined using the plaque assay and the viral stocks were stored as aliquots at −80 °C. All the SARS-CoV-2 experiments, excluding those using inactivated SARS-CoV-2, were performed in a biosafety level 3 (BSL-3) or BSL-4 laboratory.

### 2.5. Western Blot

The protein lysates of SARS-CoV-2-infected cells were dissolved with RIPA buffer. The protein expression was analyzed by Western blot analysis in which the TGN Gel 4–15% (QP5510, SMOBIO) with an SE260 Mighty Small II Deluxe Mini Vertical Protein Electrophoresis Unit (Hoefer, Inc., Holliston, MA, USA) was used. A YesBlot™ Western Marker I (10–200 kDa) (WM1000 Smobio, Taiwan) was used as a molecular weight marker. Antibodies including the mouse monoclonal anti-spike (1:5000; GeneTex Cat #GTX632604) and mouse monoclonal anti-nucleocapsid (1:2000; GeneTex Cat# GTX632269) antibodies were used to detect the SARS-CoV-2 spike and nucleocapsid proteins, respectively. The mouse monoclonal GAPDH antibody (GTX627408, GeneTex, 1:5000) was used as the loading control. The anti-chicken IgY (IgG) (whole molecule)–peroxidase antibody produced in rabbits (Sigma-Aldrich A9046) and the anti-rabbit IgG (whole molecule)–peroxidase antibody produced in goats (Sigma-Aldrich A0545) were used as the secondary antibodies as required. The luminol-based detection of Western blot signals was performed using the Amersham ECL Western Blotting Detection Kit (Amersham Biosciences, Buckinghamshire, UK). The images were captured and analyzed using Image Quant™ LAS 4000 (GE Healthcare, Chicago, IL, USA).

### 2.6. The Plaque Reduction Neutralization Test (PRNT) with Infectious SARS-CoV-2

To determine the titers of neutralizing antibodies (nAbs) from anti-S protein IgYs, IgYs were first diluted ten-fold (starting at a concentration of 10,000 μg) in the DMEM. Following the heat inactivation of the complement, 0.1 mL of the serial dilutions was incubated with a virus solution containing ~100 pfu of SARS-CoV-2 Wuhan or α strain. The mixture of IgY and SARS-CoV-2 solution was added to the culture of Vero E6 cells in 24-well plates incubated at 37 °C. The Vero E6 cells were then cultured in DMEM supplemented with 2% FBS and 0.3% agarose for 3 days. Crystal violet solution was then used for the staining of the plaques. After the staining, the plaques were counted. The titers for IgY neutralizing antibody were calculated as the reciprocal of the maximal dilution of the 50% virus titer reduction compared to the negative control mock IgY produced using the same adjuvant without antigens.

### 2.7. Determination of IgY In Vitro Specificity

The SARS-CoV-2-infected Vero E6 cells were lysed using the RIPA buffer supplemented with protease inhibitor (Merck Millipore, Billerica, MA, USA) and incubated at 100 °C for 5 min. A total of 15 μL of each sample was loaded into wells with the A YesBlot™ Western Marker I (10–200kDa) (WM1000 Smobio, Taiwan). Membranes were immunoreacted with IgYs against SARS-CoV-2 S protein subunits or glyceraldehyde 3-phosphate dehydrogenase (GAPDH) antibodies (GeneTex, Cat# GTX627408, 1:5000). Reactive bands were visualized by Amersham ECL Western Blotting Detection Kit (Amersham)

### 2.8. SARS-CoV-2 Infection in Syrian Hamsters and IgY Treatment

Owing to the expression of endogenous ACE2 expression, Syrian golden hamsters were used as the in vivo model for SARS-CoV-2 infection. The in vivo experiments were conducted as described previously [27] and approved by the Institutional Animal Care and Use Committee (IACUC) of the National Defense Medical Center Laboratory Animal Center (AN-110-15 and AN-110-16). Eight-week-old male Syrian hamsters were purchased from the National Laboratory Animal Center (Taipei, Taiwan). To infect the Syrian hamsters, 1000 pfu of SARS-CoV-2 (Wuhan strain) were delivered intranasally. IgYs specific for S1-RBD and S2 subunits were mixed and given simultaneously via the intranasal and intraperitoneal routes to neutralize SARS-CoV-2 infectivity in the nasal cavity and in the circulation. Syrian hamsters were treated with IgY antibodies 1.5 h before or after SARS-CoV-2 infection. Syrian hamsters were randomly separated into 6 groups: (A) IgY Mock; (B) IgY Mock + CoV2 (IgYs were given 1.5 h before viral infection); (C) CoV2 + IgY Mock (IgYs were given 1.5 h after viral infection); (D) IgY S1-RBD/S2; (E) IgY S1-RBD/S2 + CoV2 (IgYs were given 1.5 h before viral infection); (F) CoV2 + IgY S1-RBD/S2 (IgYs were given 1.5 h after viral infection). Each group contained at least three hamsters. After infection and the administration of IgYs, the body weight of Syrian hamsters was monitored for 7 days. On day 4 post-infection, lung sections were subjected to H&E staining and immunohistochemistry. Since the SARS-CoV-2 spike (S) protein tightly interacts with ACE2 after viral infection, this marker is seldom assessed in SARS-CoV-2-infected host cells. Syrian hamsters were challenged by SARS-CoV-2 in animal biosafety level 3 (ABSL-3) laboratories. Considering the high infectivity and biohazards of SARS-CoV-2, we only examined the changes in body weight and the expression of SARS-CoV-2 nucleocapsid, a viral protein implicated in SARS-CoV-2 replication protein after viral infection. The Syrian golden hamster model for in vivo SARS-CoV-2 infection is highly reproducible and routinely conducted in our laboratory. All animal procedures complied with the ARRIVE guidelines (https://arriveguidelines.org/ (31 December 2020) and were reviewed and approved by the Institute of Preventive Medicine, National Defense Medical Center Animal Care and Use Committee (approval numbers: AN-110-15 and AN-110-16)

### 2.9. Immunohistochemistry

After the SARS-CoV-2 infection, lung tissue from uninfected control hamsters or infected hamsters was autopsied and fixed in 4% paraformaldehyde for 48 hours. Subsequently, paraffin sections (4 μm in thickness) were made and H&E staining was used to identify the histopathological changes. In addition, immunohistochemistry staining (IHC) was used for the detection of the SARS-CoV-2 nucleocapsid protein with SARS-CoV-2 (COVID-19) nucleocapsid antibody (GeneTex, Cat #GTX135357; 1:1000). The lobe sections were obtained over the whole lung and all of the slides were subjected to the automatic sample preparation system BenchMark XT and the overview scan was performed using the VENTANA DP 200 slide scanner (Hong Jing Co., Ltd., New Taipei, Taiwan).

### 2.10. Statistical Analysis

Data were expressed as mean ± SD and analyzed using GraphPad Prism v. 6.0.1. (GraphPad Software, La Jolla, CA, USA). Statistically significant differences among groups were detected by one-way ANOVA. Once a significantly difference was detected, post hoc Tukey tests were conducted. Differences in the values between the two groups were analyzed with the unpaired Student’s two-tailed *t*-test. The criterion for significance was set at *p* < 0.05.

## 3. Results

### 3.1. Post-Immunization Profiling of IgY in Avian Serum and Egg Yolk

**Figure 1:**
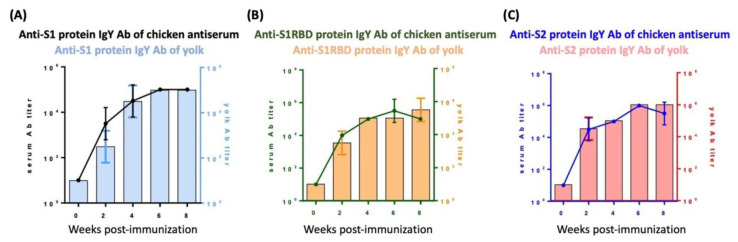
Detection of antibody titers in chicken and egg yolk anti-sera after immunization of laying hens. After immunizing laying hens with antigens derived from the S1, S1-RBD, or S2 subunits of the SARS-CoV-2 S protein, IgYs were collected from hens or egg yolks and IgY titers were specifically detected using ELISA. Increases in IgY titers for the (**A**) S1 subunit, (**B**) S1-RBD subunit, and (**C**) S2 subunit at different time points post-immunization are shown. In each panel, the line graph represents the time-course of the chicken IgY titer, and the bar graph represents the egg yolk IgY titer. Data are expressed as mean ± SD of 3 immunized hens and the egg yolk from 3 immunized eggs from each time point, respectively.

We analyzed the antibody titer of IgYs purified from either avian serum or egg yolk at various time points after immunizing laying hens. A robust increase in IgYs specific for S1, S1-RBD, and S2 subunits of SARS-CoV-2 S protein was detected two weeks after immunization in both serum and egg yolk (Figure 1A–C). IgY titers plateaued by four weeks post-immunization and were similar between the serum and egg yolk (Figure 1A–C). IgY titers remained high during the experimental period of eight weeks.

### 3.2. Yolk IgYs Specific for S protein Subunits Neutralize SARS-CoV-2 Infectivity In Vitro

**Figure 2:**
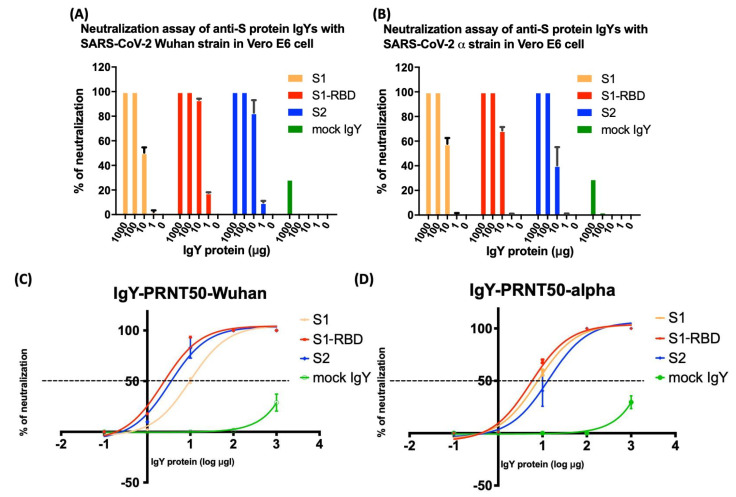
The plaque reduction neutralization test (PRNT) was used to measure the neutralizing potential of IgYs specific for S1, S1-RBD, and S2 subunits against different strains of SARS-CoV-2. Vero E6 cells were treated with a mixture of SARS-CoV-2 (Panel (**A**): Wuhan strain; Panel (**B**): Alpha strain) and different amounts of IgY proteins specific for S1, S1-RBD, S2 subunits, and mock control IgY. After treatment, viral titers were evaluated by plaque assays. PRNT50 is known as the standard method for measuring neutralizing antibodies. To assess the PRNT50 of each neutralizing antibody that can suppress 50% of infectious viruses, the antibody neutralizing curves for each SARS-CoV-2 strain were plotted (Panel (**C**): Wuhan strain; Panel (**D**): Alpha strain). Data are presented as mean ± SD of three independent experiments.

Next, we examined the potential of IgYs specific for S1, S1-RBD, and S2 subunits to neutralize viral infection by different SARS-CoV-2 strains. The plaque reduction neutralization test (PRNT), the most commonly used method for measuring neutralizing antibodies, was used to examine the neutralizing capacity of each antibody. First, 0–10 µg of IgYs specific for S1, S1-RBD, or S2 subunits was pre-mixed with 100 pfu/well of SARS-CoV-2 (Wuhan or Alpha strain) to neutralize the viral infectivity. Yolk IgY from unimmunized laying hens was used as the mock control. Vero E6 cells were exposed to the mixture of SARS-CoV-2 and IgYs specific for various S protein subunits. Viral titers were evaluated by plaque assay. Coronaviruses bind to their receptor expressed on host cells via the homotrimeric S protein, which comprises S1 and S2 subunits. The receptor-binding domain (RBD) within the S1 subunit is a pivotal functional region that governs the binding of SARS-CoV-2 to ACE2 expressed on host cells [28]. IgYs specific for the S1 subunit neutralized 50–60% of both SARS-CoV-2 strains at 10 μg/well and reached maximal neutralization at 100 μg/well (Figure 2A,B). For the SARS-CoV-2 Alpha strain, anti-S1 and anti-S1-RBD IgYs had similar neutralizing efficacies (Figure 2A). For the SARS-CoV-2 Wuhan strain, IgYs specific for the S1-RBD exhibited a higher neutralizing ability than in the Alpha strain (Figure 2B). IgYs specific for the S2 subunit led to ~40% neutralization of viral infectivity at 1 μg/well for the Alpha strain and 80–90% neutralization at the same dose for the Wuhan strain. Maximal neutralization by anti-S2 IgY was observed at 10 μg/well in both strains (Figure 2A,B). To further assess the neutralization capability of each IgY antibody, the 50% plaque reduction neutralization titer (PRNT50) of each given antibody was measured (Figure 2C,D). The definition of PRNT50 is the antibody dilutions that can suppress >50% of viral plaques. The PRNT50 of IgYs specific for S1, S1-RBD, and S2 subunits against the Wuhan strain were 9.882 μg/mL, 2.317, and 3.339 μg/mL, respectively. The PRNT50 of IgYs for S1, S1-RBD, and S2 subunits against the Alpha strain were 7.657, 5.436, and 13.82 μg/mL, respectively (Table 1). Among these neutralizing antibodies, IgY for S1-RBD has the lowest PRNT50, compared to other neutralizing antibodies. Collectively, these findings indicate that IgYs specific for S1, S1-RBD, and S2 subunits all carry the potential to neutralize viral infectivity in vitro.

### 3.3. In Vitro Specificity and Immunoreactivity of IgYs to Target Full-Length Spike Protein

**Figure 3:**
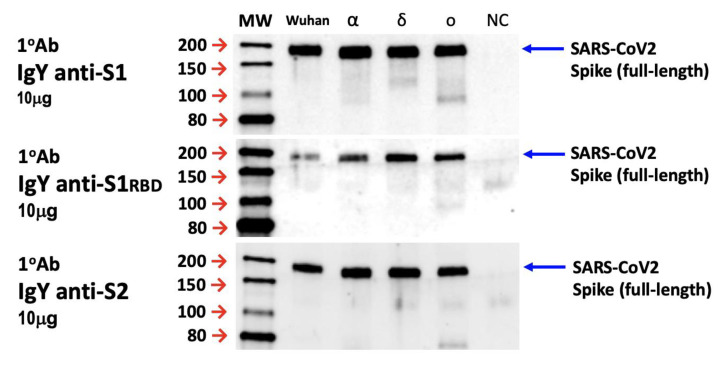
IgYs specific for S1, S1-RBD, or S2 subunits of SARS-CoV-2 (S) spike protein interact specifically with full-length spike proteins. The Vero E6 cells infected with indicated strain of SARS-CoV-2 (i.e., the Wuhan, Alpha, Delta, and Omicron strains) were harvested and protein lysates were collected. Then, 10 μg/mL of egg yolk IgYs specific for S1, S1-RBD, or S2 subunits were used as the primary antibodies to immuno-react with the protein lysates from SARS-CoV-2-infected Vero E6 cells. The specificity and immunoreactivity of the purified IgY products to spike proteins of different SARS-CoV-2 strains within the cell lysates are demonstrated.

In addition to the neutralization assays with Vero E6 cells, we examined the in vitro specificity and immunoreactivity of IgYs specific for the S1, S1-RBD, and S2 subunits to full-length SARS-CoV-2 S protein. We subjected the full-length S protein of various SARS-CoV-2 strains (Wuhan, α, δ, and ο strains) to Western blotting and used 10 μg of IgYs specific for S1, S1-RBD, or S2 subunits as primary antibodies. Remarkably, all the egg yolk IgYs were able to interact specifically with the full-length S protein derived from SARS-CoV-2 Wuhan, α, δ, and ο strains (Figure 3), demonstrating the specificity of egg yolk IgYs for SARS-CoV-2 S proteins.

### 3.4. IgY Treatment Rescues Weight Loss in SARS-CoV-2-Infected Hamsters

**Figure 4:**
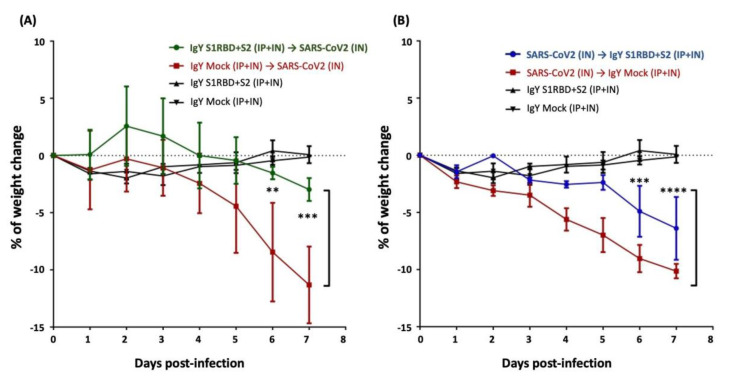
Treatment efficacy of egg yolk IgYs in SARS-CoV-2-infected Syrian hamsters. The efficacy of combination treatment of IgYs specific for S1-RBD and S2 subunits (IgY S1RBD + S2) was examined in Syrian hamsters, and mock IgY was used as the control (IgY Mock). IgYs were simultaneously delivered intranasally and intraperitoneally, either 1.5 h before (**A**) or after (**B**) intranasal delivery of the SARS-CoV-2 Wuhan strain. SARS-CoV-2 infection robustly decreased body weight within 7 days. IgY S1RBD + S2 treatment did not modify body weight in uninfected hamsters. In infected hamsters, administration of IgY S1RBD + S2 before or after infection significantly rescued the weight loss observed in infected hamsters. Data are presented as mean ± SD of three Syrian hamsters from three independent experiments. ** *p* < 0.01, *** *p* < 0.001, **** *p* < 0.0001.

Next, we sought to examine the potential of egg yolk IgYs to neutralize SARS-CoV-2 infectivity in vivo. Actually, the high infectivity of SARS-CoV-2 is a well-known factor that largely restricted the development of virology research on COVID-19. Most SARS-CoV-2 virological experiments need to be conducted in biosafety level 3 (BSL-3) and animal biosafety level 3 (ABSL-3) laboratories. A platform that mimics the SARS-CoV-2 pathogenesis in the human body is critical for investigating SARS-CoV-2 virology in the host. Since human and Syrian golden hamster ACE2 share high sequence similarity and Syrian hamsters have been shown to be susceptible to SARS-CoV-2 infection, Syrian golden hamsters can serve as an in vivo model for SARS-CoV-2 infection for the examination of vaccination efficacy and therapeutics [27,29,30]. The major phenotype of Syrian golden hamsters in response to SARS-CoV-2 infection is mild weight loss which spontaneously recovers at two weeks post-infection (Imai M. et al. Proc Acad Sci U S A. 2020 Jul 14;117(28):16587-16595.). Therefore, following these backgrounds, we also used the changes in body weight as a major indicator of disease manifestations after SARS-CoV-2 infection in this study. We delivered IgYs specific for S1-RBD and S2 via the intranasal and intraperitoneal routes simultaneously to neutralize the S1 receptor-binding domain and the S2 subunit in SARS-CoV-2-infected Syrian hamsters. The delivery of IgYs was conducted 1.5 h before or after intranasal challenge with 1000 pfu of SARS-CoV-2 (Wuhan strain). Administration of mock IgY or the combination of IgYs specific for S1-RBD and for S2 did not modify baseline body weight (Figure 4A,B). Intranasal challenge with SARS-CoV-2 led to a consistent reduction in body weight of 10% within 7 days. Mock IgY delivered before or after SARS-CoV-2 infection showed no effect on this reduction in body weight. Pre-treatment with IgYs specific for S1-RBD and S2 subunits prevented the robust SARS-CoV-2-induced reduction of body weight, resulting in only ~3% weight loss by the end of the 7-day experimental period. Additionally, treatment with IgYs specific for S1-RBD and S2 subunits after SARS-CoV-2 challenge also significantly rescued the weight loss from 10% to 6% (Figure 4A,B).

### 3.5. IgYs Exhibit Marked Efficacy in Both Prophylaxis and Treatment of SARS-CoV-2 Infection

**Figure 5:**
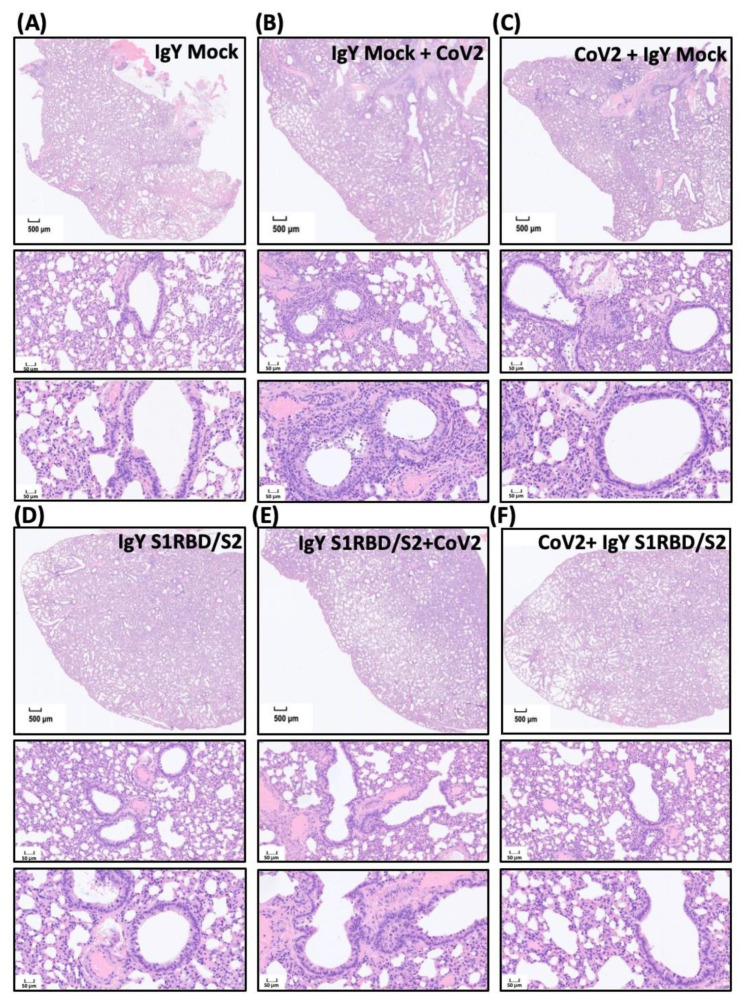

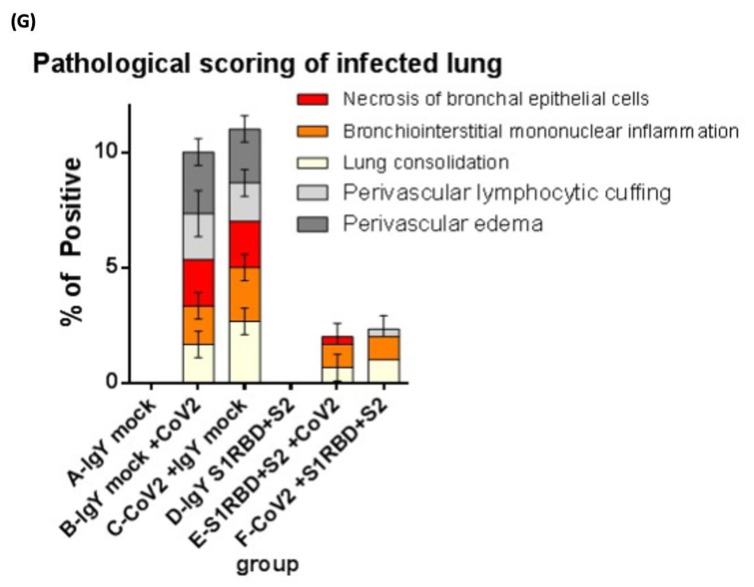
Effect of IgYs specific for S1-RBD and S2 subunits on pathological changes in SARS-CoV-2-infected lung tissues in vivo. The efficacy of treatment with the combination of IgYs specific for S1-RBD and S2 subunits (IgY S1RBD + S2) was examined in Syrian hamsters, and IgY from mock infection was used as the control (IgY Mock). IgYs were simultaneously delivered intranasally and intraperitoneally, either 1.5 h before or after the intranasal delivery of the SARS-CoV-2 Wuhan strain. Four days after SARS-CoV-2 infection, infected hamsters were sacrificed and the lung tissues from hamsters with indicated treatment were collected. Pathological changes in lung sections from Syrian hamsters with indicated IgY pre-treatments are shown (Panels (**A**–**F**)). Results of H&E staining are presented using representative images of three independent experiments. Images of H&E staining with lower (the upper subpanel in each panel, scale bar = 500 μm) and higher magnification (the middle and lower subpanels in each panel, scale bar = 50 μm) are shown. In panels (**A**,**D**), homogeneous interstitial and alveolar spaces are noted. In panels (**B**,**C**), various high cellularity patches show inflammatory cell infiltration around bronchioles proximal to the bronchus. In panel (**B**), remarkable lymphocytic–neutrophilic infiltration is observed; in panel (**C**), the necrotic changes of the bronchial lumen epithelium, lymphocytic adhesion to endothelium, and perivascular edema are noted. In panel (**G**), the pathological score based on various parameters, including necrosis of bronchial epithelial cells, bronchiointerstitial mononuclear inflammation, lung consolidation, perivascular lymphocytic cuffing, and perivascular edema, is calculated.

Next, we evaluated SARS-CoV-2-induced lesions and treatment efficacy of IgY antibodies in vivo. Mock IgY treatment alone had no effect on lung section morphology, as observed by hematoxylin and eosin (H&E) staining (Figure 5A). IgYs were delivered 1.5 h before or after intranasal challenge with SARS-CoV-2 (1000 pfu, Wuhan strain). Intranasal delivery of SARS-CoV-2 resulted in multifocal to coalescent areas of consolidation in lung sections, accompanied by inflammatory cell infiltration. In addition, neutrophil, macrophage, and lymphocyte infiltration were mainly found in perivascular areas. Pre-treatment (Figure 5B) or treatment with mock IgY after viral infection (Figure 5C) did not modify the pathological changes observed in the lungs of Syrian hamsters (Figure 5B,C). The patchy distribution of high cellularity indicating inflammatory cell infiltration is seen around bronchioles proximal to the bronchus in groups B and C. Significant lymphocytic–neutrophilic infiltration (Figure 5B) and perivascular edema (Figure 5C) are found under a high-power field with neutrophilic adhesion to the bronchial epithelium within the lumen (Figure 5B). Necrotic changes of epithelium and cell debris within the bronchial lumen are noted (Figure 5C). Lymphocytic adhesion to endothelium and perivascular edema suggest vasculitis (Figure 5C). The interstitial and alveolar space of groups A and D appears homogeneous. As per the body weight data, intranasal and intraperitoneal delivery of IgYs specific for S1-RBD and S2 subunits in uninfected hamsters did not induce any detectable histological changes (Figure 5D). Notably, pre-treatment (Figure 5E) or treatment (Figure 5F) with a combination of IgYs specific for S1-RBD and S2 subunits ameliorated the pulmonary insults caused by SARS-CoV-2 infection (Figure 5E,F). Meanwhile, we also requested the assistance of a pathologist to quantify these pathological lesions and calculate the pathological score based on various parameters, including necrosis of bronchial epithelial cells, bronchiointerstitial mononuclear inflammation, lung consolidation, perivascular lymphocytic cuffing, and perivascular edema (Figure 5G). The SARS-CoV-2 infection prominently increased indicated pathological scores which were not affected by IgY Mock pre-treatment nor post-treatment. Pre-treatment or treatment of the combination of IgYs specific for S1-RBD and S2 subunits robustly reduced lung pathological changes (Figure 5G).

**Figure 6:**
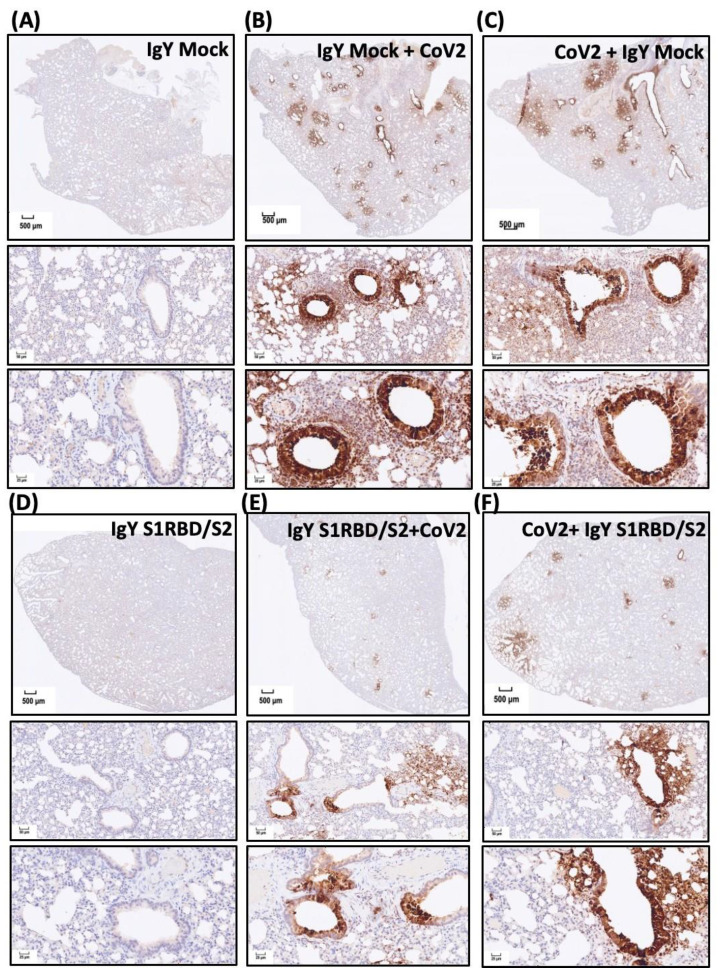

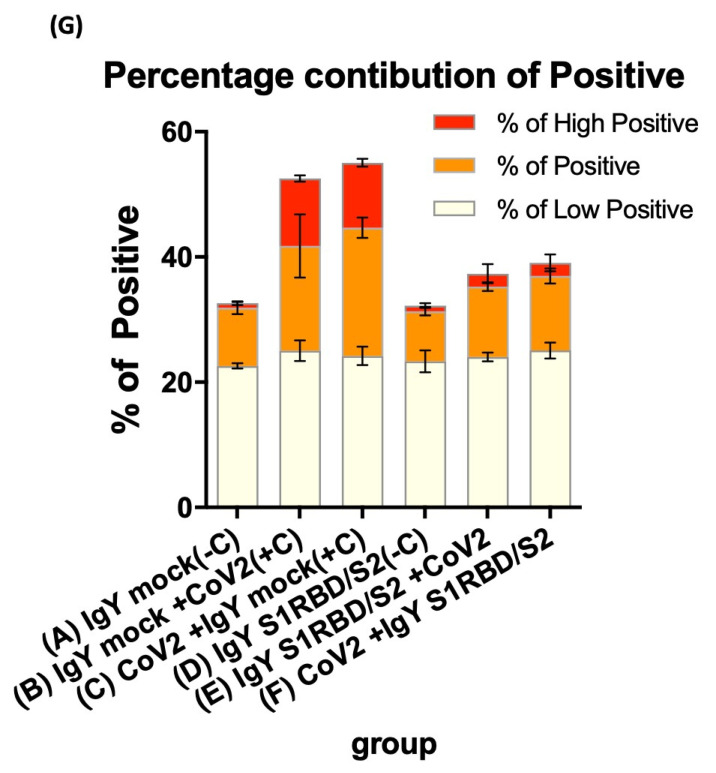
Effect of treatment with IgYs specific for S1-RBD and S2 subunits on SARS-CoV-2 infection in vivo. Infected regions were visualized by immunohistochemistry staining of the SARS-CoV-2 nucleocapsid (N) protein (a SARS-CoV-2 infection marker). The efficacy of the combination of IgYs specific for S1-RBD and S2 subunits (IgY S1RBD + S2) was examined in Syrian hamsters, and mock IgY was used as the control (IgY Mock). IgYs were simultaneously delivered intranasally and intraperitoneally, either 1.5 h before or after intranasal delivery of the SARS-CoV-2 Wuhan strain. Four days after SARS-CoV-2 infection, infected hamsters were sacrificed and the lung tissues from hamsters with indicated treatment were collected. Lung areas positive for SARS-CoV-2 N protein staining in Syrian hamsters with indicated IgY pre-treatments are shown (Panels (**A**–**F**)). In panel (**G**), the immunoreactive N protein-positive cells are quantified and classified into three groups, i.e., high-positive, positive, and low-positive groups, to show the magnitude of SARS-CoV-2 replication in the hosts. Results of immunohistochemistry staining are presented using representative images of three independent experiments.

To validate the findings regarding the SARS-CoV-2-infected areas and to examine the treatment efficacy of IgYs specific for S1-RBD and S2 subunits, we subjected lung sections from hamsters with the indicated treatments to immunohistochemical staining (Figure 6A–F). Infected regions were visualized by staining SARS-CoV-2 nucleocapsid (N) protein, a SARS-CoV-2 infection marker. Treatment with mock IgY or the combination of IgYs specific for S1-RBD or S2 subunits did not induce N protein signal in uninfected lung sections (Figure 6A,D). Multifocal regions positive for N protein staining were ubiquitous across the infected lung tissue from hamsters pre-treated or treated with mock IgY (Figure 6B,C). In infected tissue, nucleocapsid-positive cells were predominantly located in the perivascular areas (Figure 6B,C). Remarkably, pre-treatment with the combination of IgYs specific for S1-RBD and S2 subunits largely prevented the increase in SARS-CoV-2 nucleocapsid-positive areas (Figure 6E). Administration of IgYs specific for S1-RBD and S2 subunits 1.5 h after SARS-CoV-2 infection also prevented the increase in SARS-CoV-2 nucleocapsid-positive areas (Figure 6F). To visualize the N protein levels in the results of immunohistochemistry, we quantified the immunoreactive N protein-positive cells and classified them into three groups, i.e., high-positive, positive, and low-positive groups. SARS-CoV-2 infection consistently increased the amount of immunoreactive N protein-positive cells which were not modified by the pre- or post-treatment of IgY Mock. The combination of IgYs specific for S1RBD and S2 subunits per se showed no obvious effect on the amount of N protein-positive cells. Pre-treatment or post-treatment of the combination of IgYs specific for S1RBD and S2 subunits both reduced the amount of N protein-positive cells (Figure 6G). These data indicated that the administration of IgY antibodies for S1RBD and S2 subunits suppressed SARS-CoV-2 replication in the infected Syrian golden hamsters. Together, both pre-treatment and post-infection treatment exhibited promising efficacy in prophylaxis and treatment of SARS-CoV-2 infection in vivo.

## 4. Discussion

The COVID-19 pandemic represents a great threat to public health and the global economy. Accordingly, numerous efforts in the development of innovative strategies to overcome this challenge have been made. Passive immunization is one of these novel therapeutic options that may hold promise in pandemic control [11]. Although monoclonal antibodies are accepted to be the best type of antibodies generated for passive immunization, they are also time-consuming and expensive to make [14]. In contrast, IgYs have drawn substantial attention due to their high functional similarities to mammalian IgG, ideal bioavailability, ease of high-yield production, and low production cost [16]. Numerous studies have demonstrated the treatment efficacy of IgY against bacterial and viral infections [20,21,22,23,24]. The SARS-CoV-2 S protein consists of a signal peptide located on the N-terminus, an S1 subunit, and an S2 subunit [31]. The S1 subunit consists of an N-terminal domain and an RBD, and the S2 subunit comprises a fusion peptide, heptapeptide repeat sequence 1, heptapeptide repeat sequence 2, a transmembrane domain, and a cytoplasmic domain. The RBD of the S1 subunit is responsible for the recognition and binding to the host ACE2 receptor and the S2 subunit governs membrane fusion [31]. In the present study, we immunized laying hens and generated egg yolk IgYs specific for the S1, S1-RBD, and S2 subunits of the SARS-CoV-2 S protein. Egg yolk IgY titers increased in a time-dependent manner in parallel with the elevation of serum IgY titers in immunized hens (Figure 1).

In addition to the characterization of IgY features, we further evaluated the in vitro specificity of IgYs and their ability to neutralize viral infectivity. IgYs specific for each S protein subunit showed excellent in vitro specificity for full-length spike protein derived from SARS-CoV-2 Wuhan, α, δ, or ο strains (Figure 3). To examine the neutralization potential of these IgYs in vitro, we pre-mixed IgYs with different SARS-CoV-2 strains and assessed viral titers using plaque assays. Notably, IgY antibodies specific for S1 and S1-RBD subunits and those specific for the S2 subunit were able to potently neutralize viral infectivity (Figure 2). A bioinformatic study using phylogenetic analysis and 3D structural modeling revealed that the SARS-CoV-2 S protein is highly conserved across variants. From the NCBI database, the identity of the overall percent protein sequence of the SARS-CoV-2 spike protein sequences was 99.68. The S1 subunit was more conserved than the S2 subunit (99.70% versus 99.66%). Based on the NCBI database of the infected countries, the 319-541 residues of amino acids within the S1 domain were 100% similar among the collected SARS-CoV-2 S protein sequences. Therefore, the SARS-CoV-2 S1-RBD subunit has been widely used as a target region for the development of vaccines [32]. Interestingly, IgY targeting the S2 subunit, a component that mediates membrane fusion, also fully neutralized viral infectivity at 100 μg. These data revealed that, similar to IgYs specific for S1 or S1-RBD subunits, IgYs that specifically target the spike protein S2 subunit may also possess the potential to neutralize the SARS-CoV-2 viral infectivity.

IgYs specific for S1, S1-RBD, and S2 subunits all exhibited the ability to neutralize SARS-CoV-2 infectivity in vitro. We examined the combined treatment efficacy of IgY specific for the S1-RBD subunit and IgY specific for the S2 subunit before or after SARS-CoV-2 infection in Syrian hamsters, an established in vivo experimental model for SARS-CoV-2 infection. Antibodies were delivered via both intranasal and intraperitoneal routes. Intranasal IgYs were administered to neutralize intranasally delivered SARS-CoV-2 and intraperitoneal injection of IgYs was employed to suppress systemic viremia induced by SARS-CoV-2 infection. The combined treatment with IgYs specific for S1-RBD and S2 subunits rescued the weight loss phenotype induced by SARS-CoV-2 infection and reduced the size of lung lesions and areas positive for SARS-CoV-2 N protein staining (Figure 4, Figure 5 and Figure 6). Pre-treatment with IgYs targeting S protein subunits restored body weight to a greater extent than treatment with these IgYs after SARS-CoV-2 infection. In addition, pre-treatment with these IgYs was more effective than post-infection treatment at reducing lung lesions and areas positive for SARS-CoV-2 nucleocapsid staining. It is encouraging that the IgY treatment after viral infection remains efficacious to rescue body weight loss and intrapulmonary lesions. In infected animals, SARS-CoV-2 may lead to secondary viral infection after viral replication and subsequently damage host cells. It is possible that post-infection treatment with IgYs specific for different SARS-CoV-2 subunits ameliorated the disease phenotype by suppressing secondary viral infections. Alternatively, pre-treatment with IgYs specific for different SARS-CoV-2 subunits may neutralize viral infectivity before cells are infected by SARS-CoV-2, leading to better outcomes.

## 5. Conclusions

IgYs have drawn attention in the field of passive immunization and vaccine development due to being inexpensive and effective antibodies that can be generated in high amounts. Supporting the utility of IgYs in COVID-19 treatment, IgYs specific for the SARS-CoV-2 S protein were shown to suppress its binding to ACE2 and viral replication in host cells [26]. We generated IgYs that specifically target different SARS-CoV-2 subunits and exhibited ideal in vitro specificity to interact with full-length spike proteins from different SARS-CoV-2 strains and had the ability to neutralize SARS-CoV-2 infectivity in vitro. Our in vivo findings indicated that pre-treatment with IgYs specific for the S1-RBD and S2 subunits showed promising efficacy to ameliorate pathogenicity and lung infection. The findings of our in vivo experiments should be of high scientific merit. However, the high biohazards and infectivity of our experiments and limited facilities made it very difficult to conduct various experiments that might cause contamination and threaten laboratory manpower. Hindered by the aforementioned limitations and considerations, we did not go deep into the evaluation of the effect of IgY administration on the hemogram and the detailed immune responses in infected Syrian hamsters. A previously published paper demonstrated that the Fc portion of IgY is unable to activate the human complement and to bind to rheumatoid factor and to protein G. Owing to structural differences and phylogenetic distance, IgY does not react with certain components of the human immune system and exhibits higher avidity for mammalian conserved proteins. Therefore, IgYs are suggested to be more suitable for diagnostic purposes than mammalian antibodies and have been extensively used as therapeutic and diagnostic tools in health research, including applications in human and veterinary health [33]. Collectively, these findings supported the reliability, biosafety, and bioavailability of IgY technologies in clinical uses in both human and veterinary health. However, more pre-clinical animal or human studies are still needed to elucidate the immunogenic issue of IgYs in detail.

## Figures and Tables

**Table 1:** PRNT_50_ of the indicated strain of SARS-CoV-2.

	Anti-S1 IgY	Anti-S1-RBD IgY	Anti-S2 IgY
PRNT_50_ of Wuhan strain (μg)	9.882	2.317	3.339
PRNT_50_ of α strain (μg)	7.657	5.436	13.82

## Data Availability

No applicable.
